# Racemic methyl 3,10-dioxa-2-aza­tri­cyclo­[6.2.1.0^2,6^]undecane-4-carboxyl­ate

**DOI:** 10.1107/S160053681101484X

**Published:** 2011-04-29

**Authors:** Basem A. Moosa, Atif Fazal, Shaikh A. Ali, Mohammed Fettouhi

**Affiliations:** a Controlled Release and Delivery Laboratory, King Abdullah University of Science and Technology, Thuwal, Saudi Arabia; b Center of Research Excellence in Petroleum Refining, and Petrochemicals Research Institute, King Fahd University of Petroleum and Minerals, Dhahran 31261, Saudi Arabia; c Department of Chemistry, King Fahd University of Petroleum and Minerals, Dhahran 31261, Saudi Arabia

## Abstract

The structure of the racemic title compound, C_10_H_15_NO_4_, consists of a tricyclic skeleton comprising a six-membered piperidine ring and five-membered isoxazolidine and tetra­hydro­furan rings. The piperidine ring adopts a distorted chair conformation, while the isoxazolidine and tetra­hydro­furan rings have envelope conformations.

## Related literature

For related piperidine geometry, see: Parkin *et al.* (2004 ▶). For bicyclic polyhydro­isoxazolopyridines, see: Banerji *et al.* (2006 ▶); Carmona *et al.* (2009 ▶). For literature related to cyclo­addition reactions of cyclic nitro­nes, see: Ali & Wazeer (1988 ▶); Ali *et al.* (1988 ▶); Merino (2004 ▶); Chandrasekhar (2005 ▶); Moosa & Ali (2009 ▶, 2010 ▶). For the natural product SB-219383 and its inhibitory activity against tyrosyl tRNA sythetase, see: Houge-Frydrych *et al.* (2000 ▶); Stefanska *et al.* (2000 ▶).
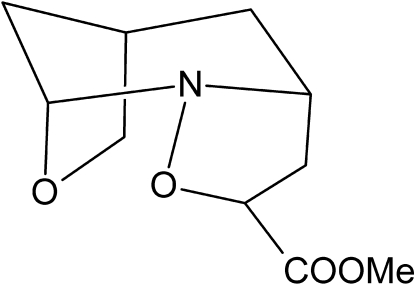


## Experimental

### 

#### Crystal data

C_10_H_15_NO_4_
*M* *_r_* = 213.23
Monoclinic, 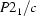

*a* = 11.213 (3) Å
*b* = 7.1075 (18) Å
*c* = 12.910 (3) Å
β = 91.546 (5)°
*V* = 1028.4 (4) Å^3^
*Z* = 4
Mo *K*α radiation
μ = 0.11 mm^−1^
*T* = 294 K
0.20 × 0.10 × 0.05 mm

#### Data collection

Bruker SMART APEX area-detector diffractometer
Absorption correction: multi-scan (*SADABS*; Sheldrick, 1996 ▶) *T*_min_ = 0.979, *T*_max_ = 0.995
13498 measured reflections
2563 independent reflections
1567 reflections with *I* > 2σ(*I*)
*R*_int_ = 0.052

#### Refinement

*R*[*F* ^2^ > 2σ(*F* ^2^)] = 0.057
*wR*(*F* ^2^) = 0.155
*S* = 1.03
2563 reflections
185 parameters
H atoms treated by a mixture of independent and constrained refinement
Δρ_max_ = 0.24 e Å^−3^
Δρ_min_ = −0.22 e Å^−3^

### 

Data collection: *SMART* (Bruker, 2008 ▶); cell refinement: *SAINT* (Bruker, 2008 ▶); data reduction: *SAINT*; program(s) used to solve structure: *SHELXS97* (Sheldrick, 2008 ▶); program(s) used to refine structure: *SHELXL97* (Sheldrick, 2008 ▶); molecular graphics: *ORTEP-3* (Farrugia, 1997 ▶); software used to prepare material for publication: *SHELXTL* (Sheldrick, 2008 ▶).

## Supplementary Material

Crystal structure: contains datablocks I, global. DOI: 10.1107/S160053681101484X/om2422sup1.cif

Structure factors: contains datablocks I. DOI: 10.1107/S160053681101484X/om2422Isup2.hkl

Supplementary material file. DOI: 10.1107/S160053681101484X/om2422Isup3.mol

Supplementary material file. DOI: 10.1107/S160053681101484X/om2422Isup4.cml

Additional supplementary materials:  crystallographic information; 3D view; checkCIF report

## Figures and Tables

**Fig. 1.**
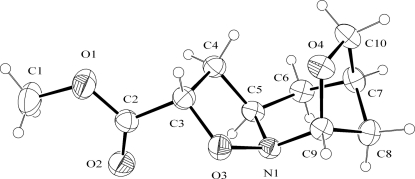
The molecular structure with atom labels and 30% probability displacement ellipsoids for non-H atoms.

## supplementary crystallographic information

### Comment

1,3-Dipolar cycloaddition reaction of cyclic nitrones with alkenes shows greater stereoselectivity and reactivity compared to their acyclic counterparts for applications in the synthesis of natural products (Merino, 2004; Moosa & Ali, 2009, 2010; Ali *et al.*, 1988; Ali & Wazeer, 1988). Our interest in developing a synthetic methodology to construct the ring skeleton present in a natural product called SB-219383 (Houge-Frydrych *et al.*, 2000), first member of a new class of compounds having inhibitory activity against tyrosyl tRNA sythetase (Stefanska *et al.*, 2000), led to explore the synthesis and cycloaddition of the bicyclic nitrone 1-oxa-5,6-dehydro-6-aza-bicyclo[3,2,1]heptane 6-oxide with methyl acrylate. The structure of a recemic sample of the cycloadduct is reported here. It consists of a tricyclic skeleton corresponding to a *cis*-invertomer with the nitrogen lone pair in axial position. The two C—N bond lengths of the piperidine ring are N1—C9: 1.453 (2) Å and N1—C5: 1.473 (2) Å. The former bond is shorter presumably as a result of anomeric effect (Chandrasekhar, 2005), inducing a partial double bond character. The bond distances are consistent with those reported for piperidine (Parkin *et al.*, 2004) and bicyclic polyhydroisoxazolopyridines (Banerji *et al.*, 2006; Carmona *et al.*, 2009). The piperidine ring adopts a distorted chair conformation likely due to the strain of the 5-membered rings. Angular constraints imposed by the five-membered tetrahydrofuran ring skeleton led to the squeezing of the C7—C8—C9 angle to 97.8 (2)°; the corresponding bond angle in piperidine, the parent six-membered heterocyclic, is 110.21 (7)°. The angles C5—N1—C9, N1—C5—C6 and C5—C6—C7 on the other end of the six-membered ring were expanded to 114.1 (2)°, 112.3 (2)° and 113.7 (2)°, respectively, while the corresponding angles in piperidine are 111.04 (7)°, 109.84 (7)° and 110.70 (7)° (Parkin *et al.*, 2004). The destabilizing diaxial interactions among the three axially oriented substituents in the piperidine ring is somewhat relieved by moving outward from their ideal axial positions. This leads to a flattening of the chair at N1, C5 and C6. The isoxazolidine ring adopts an envelope conformation with N1, O3, C3 and C4 essentially in the plane while C5 is 0.482 (3) Å out of the plane. The dihedral angle between the previous plane and the plane N1—C5—C4 is 31.9 (2)°. The tetrahydrofuran ring has also an envelope conformation with C8 at 0.733 (3) Å out of the plane C9—O4—C10—C7 which has a dihedral angle of 47.0 (2)° with the plane C7—C8—C9.

### Experimental

To a stirred solution of *N*-hydroxy-4-piperidinemethanol (0.39 g, 3.0 mmol) in chloroform (20 ml) was added mercuric oxide (0.65 g, 12 mmol) and anhydrous magnesium sulfate (150 mg) in 10 min. The reaction mixture was then stirred at room temperature for 6 h. The mixture was filtered over a bed of magnesium sulfate to obtain a solution of the bicyclic nitrone 1-oxa-5,6-dehydro-6-aza-bicyclo[3,2,1]heptane 6-oxide which was used without isolation. The solution of the nitrone and methyl acrylate (2 ml) was stirred at room temperature for 4 h. After removal of the solvent and excess methyl acrylate, the reaction mixture was concentrated and the residual liquid was chromatographed over silica using 9:1 ether/methanol mixture as an eluant to give the cycloadduct as a solid (0.28 g, 44%). Colorless blocks were obtained after crystallization at 0°C from ether/CH_2_Cl_2_ (9/1) mixture.

### Refinement

Methyl H atoms were included as a rigid group and refined using a riding model with C—H = 0.96 Å and *U*_iso_(H) = 1.5*U*_eq_(C). All other H atoms were located in a difference Fourier map and refined isotropically.

### Crystal data

**Table:**

C_10_H_15_NO_4_	*F*(000) = 456
*M**_r_* = 213.23	*D*_x_ = 1.377 Mg m^−^^3^
Monoclinic, *P*2_1_/*c*	Mo *K*α radiation, λ = 0.71073 Å
Hall symbol: -P 2ybc	Cell parameters from 13498 reflections
*a* = 11.213 (3) Å	θ = 1.8–28.4°
*b* = 7.1075 (18) Å	µ = 0.11 mm^−^^1^
*c* = 12.910 (3) Å	*T* = 294 K
β = 91.546 (5)°	Block, colourless
*V* = 1028.4 (4) Å^3^	0.20 × 0.10 × 0.05 mm
*Z* = 4	

### Data collection

**Table:**

Bruker SMART APEX area-detector diffractometer	2563 independent reflections
Radiation source: normal-focus sealed tube	1567 reflections with *I* > 2σ(*I*)
graphite	*R*_int_ = 0.052
ω scans	θ_max_ = 28.4°, θ_min_ = 1.8°
Absorption correction: multi-scan (*SADABS*; Sheldrick, 1996)	*h* = −14→14
*T*_min_ = 0.979, *T*_max_ = 0.995	*k* = −9→9
13498 measured reflections	*l* = −17→17

### Refinement

**Table:**

Refinement on *F*^2^	Primary atom site location: structure-invariant direct methods
Least-squares matrix: full	Secondary atom site location: difference Fourier map
*R*[*F*^2^ > 2σ(*F*^2^)] = 0.057	Hydrogen site location: inferred from neighbouring sites
*wR*(*F*^2^) = 0.155	H atoms treated by a mixture of independent and constrained refinement
*S* = 1.03	*w* = 1/[σ^2^(*F*_o_^2^) + (0.0701*P*)^2^ + 0.2128*P*] where *P* = (*F*_o_^2^ + 2*F*_c_^2^)/3
2563 reflections	(Δ/σ)_max_ = 0.002
185 parameters	Δρ_max_ = 0.24 e Å^−^^3^
0 restraints	Δρ_min_ = −0.22 e Å^−^^3^

### Special details

**Table:**

Geometry. All e.s.d.'s (except the e.s.d. in the dihedral angle between two l.s. planes) are estimated using the full covariance matrix. The cell e.s.d.'s are taken into account individually in the estimation of e.s.d.'s in distances, angles and torsion angles; correlations between e.s.d.'s in cell parameters are only used when they are defined by crystal symmetry. An approximate (isotropic) treatment of cell e.s.d.'s is used for estimating e.s.d.'s involving l.s. planes.
Refinement. Refinement of *F*^2^ against ALL reflections. The weighted *R*-factor *wR* and goodness of fit *S* are based on *F*^2^, conventional *R*-factors *R* are based on *F*, with *F* set to zero for negative *F*^2^. The threshold expression of *F*^2^ > σ(*F*^2^) is used only for calculating *R*-factors(gt) *etc*. and is not relevant to the choice of reflections for refinement. *R*-factors based on *F*^2^ are statistically about twice as large as those based on *F*, and *R*-factors based on ALL data will be even larger.

### Fractional atomic coordinates and isotropic or equivalent isotropic displacement parameters (Å^2^)

**Table:**

	*x*	*y*	*z*	*U*_iso_*/*U*_eq_	
N1	0.14231 (14)	0.9997 (2)	0.39231 (12)	0.0433 (4)	
O1	0.43183 (15)	0.7626 (3)	0.60910 (14)	0.0821 (6)	
O2	0.24093 (17)	0.6880 (3)	0.59172 (15)	0.0838 (6)	
O3	0.23751 (14)	0.86158 (19)	0.40074 (11)	0.0559 (4)	
O4	0.26276 (13)	1.22561 (19)	0.29897 (11)	0.0516 (4)	
C1	0.4428 (3)	0.6375 (5)	0.6972 (2)	0.0950 (11)	
H1A	0.3834	0.6687	0.7465	0.142*	
H1B	0.5207	0.6507	0.7290	0.142*	
H1C	0.4316	0.5099	0.6745	0.142*	
C2	0.3263 (2)	0.7726 (3)	0.56322 (17)	0.0514 (5)	
C3	0.3243 (2)	0.9186 (3)	0.47749 (16)	0.0484 (5)	
C4	0.2854 (2)	1.1087 (3)	0.51816 (18)	0.0508 (5)	
C5	0.15285 (19)	1.1108 (3)	0.48841 (15)	0.0458 (5)	
C6	0.0935 (2)	1.3043 (3)	0.47755 (17)	0.0515 (5)	
C7	0.1024 (2)	1.3900 (3)	0.36965 (17)	0.0535 (6)	
C8	0.0548 (2)	1.2468 (3)	0.29092 (19)	0.0542 (6)	
C9	0.15798 (18)	1.1088 (3)	0.29848 (15)	0.0441 (5)	
C10	0.2287 (2)	1.4113 (3)	0.3337 (2)	0.0565 (6)	
H4	0.400 (2)	0.929 (3)	0.4479 (16)	0.056 (6)*	
H5	0.3284 (19)	1.202 (3)	0.4850 (17)	0.050 (6)*	
H6	0.301 (2)	1.116 (3)	0.590 (2)	0.069 (7)*	
H7	0.1119 (18)	1.042 (3)	0.5399 (16)	0.049 (6)*	
H8	0.011 (2)	1.290 (3)	0.4920 (18)	0.053 (6)*	
H9	0.1310 (19)	1.383 (3)	0.5271 (17)	0.051 (6)*	
H10	0.061 (2)	1.508 (4)	0.3653 (17)	0.061 (6)*	
H11	−0.020 (2)	1.189 (3)	0.3108 (17)	0.060 (7)*	
H12	0.050 (2)	1.304 (4)	0.220 (2)	0.073 (7)*	
H13	0.1645 (16)	1.018 (3)	0.2423 (15)	0.041 (5)*	
H14	0.235 (2)	1.501 (4)	0.2774 (19)	0.072 (7)*	
H15	0.284 (2)	1.451 (3)	0.3903 (18)	0.056 (6)*	

### Atomic displacement parameters (Å^2^)

**Table:**

	*U*^11^	*U*^22^	*U*^33^	*U*^12^	*U*^13^	*U*^23^
N1	0.0489 (10)	0.0403 (9)	0.0410 (9)	−0.0017 (7)	0.0035 (7)	−0.0061 (7)
O1	0.0499 (10)	0.1099 (15)	0.0864 (13)	−0.0060 (9)	−0.0012 (9)	0.0487 (11)
O2	0.0686 (12)	0.0947 (14)	0.0873 (13)	−0.0279 (11)	−0.0100 (10)	0.0354 (11)
O3	0.0732 (11)	0.0427 (8)	0.0513 (9)	0.0081 (7)	−0.0086 (7)	−0.0092 (7)
O4	0.0545 (9)	0.0480 (8)	0.0532 (9)	−0.0020 (7)	0.0173 (7)	−0.0050 (7)
C1	0.0693 (18)	0.124 (3)	0.091 (2)	0.0066 (17)	−0.0051 (15)	0.058 (2)
C2	0.0501 (13)	0.0510 (12)	0.0534 (13)	−0.0031 (10)	0.0046 (10)	0.0037 (10)
C3	0.0473 (12)	0.0519 (12)	0.0462 (12)	−0.0021 (10)	0.0027 (9)	0.0016 (9)
C4	0.0619 (14)	0.0484 (12)	0.0420 (12)	−0.0078 (11)	−0.0028 (10)	−0.0022 (10)
C5	0.0555 (13)	0.0459 (11)	0.0363 (10)	−0.0072 (9)	0.0093 (9)	−0.0032 (9)
C6	0.0563 (14)	0.0519 (12)	0.0470 (12)	0.0013 (11)	0.0122 (10)	−0.0102 (10)
C7	0.0668 (15)	0.0436 (11)	0.0505 (12)	0.0114 (11)	0.0099 (10)	−0.0018 (10)
C8	0.0569 (14)	0.0598 (14)	0.0460 (13)	0.0079 (11)	0.0021 (11)	−0.0017 (10)
C9	0.0495 (12)	0.0448 (11)	0.0382 (11)	0.0015 (9)	0.0059 (9)	−0.0069 (9)
C10	0.0726 (16)	0.0437 (12)	0.0539 (14)	−0.0049 (11)	0.0158 (12)	0.0010 (10)

### Geometric parameters (Å, °)

**Table:**

N1—O3	1.452 (2)	C4—H5	0.93 (2)
N1—C9	1.453 (2)	C4—H6	0.94 (3)
N1—C5	1.473 (2)	C5—C6	1.532 (3)
O1—C2	1.311 (3)	C5—H7	0.95 (2)
O1—C1	1.446 (3)	C6—C7	1.526 (3)
O2—C2	1.197 (3)	C6—H8	0.95 (2)
O3—C3	1.429 (3)	C6—H9	0.94 (2)
O4—C9	1.439 (2)	C7—C10	1.509 (3)
O4—C10	1.449 (3)	C7—C8	1.525 (3)
C1—H1A	0.9600	C7—H10	0.96 (2)
C1—H1B	0.9600	C8—C9	1.518 (3)
C1—H1C	0.9600	C8—H11	0.98 (2)
C2—C3	1.517 (3)	C8—H12	1.01 (3)
C3—C4	1.518 (3)	C9—H13	0.976 (19)
C3—H4	0.95 (2)	C10—H14	0.97 (3)
C4—C5	1.525 (3)	C10—H15	0.99 (2)
			
O3—N1—C9	108.55 (14)	C6—C5—H7	107.9 (12)
O3—N1—C5	104.91 (14)	C7—C6—C5	113.74 (17)
C9—N1—C5	114.04 (16)	C7—C6—H8	107.9 (14)
C2—O1—C1	116.43 (19)	C5—C6—H8	108.0 (13)
C3—O3—N1	110.24 (14)	C7—C6—H9	110.1 (13)
C9—O4—C10	107.76 (15)	C5—C6—H9	106.8 (13)
O1—C1—H1A	109.5	H8—C6—H9	110.3 (19)
O1—C1—H1B	109.5	C10—C7—C8	100.20 (18)
H1A—C1—H1B	109.5	C10—C7—C6	113.9 (2)
O1—C1—H1C	109.5	C8—C7—C6	108.17 (19)
H1A—C1—H1C	109.5	C10—C7—H10	110.8 (13)
H1B—C1—H1C	109.5	C8—C7—H10	112.4 (14)
O2—C2—O1	123.6 (2)	C6—C7—H10	110.9 (13)
O2—C2—C3	124.9 (2)	C9—C8—C7	97.78 (18)
O1—C2—C3	111.22 (19)	C9—C8—H11	111.8 (14)
O3—C3—C2	107.94 (18)	C7—C8—H11	113.3 (13)
O3—C3—C4	107.17 (17)	C9—C8—H12	110.2 (14)
C2—C3—C4	110.81 (18)	C7—C8—H12	110.5 (15)
O3—C3—H4	110.3 (13)	H11—C8—H12	112 (2)
C2—C3—H4	110.7 (13)	O4—C9—N1	114.92 (16)
C4—C3—H4	109.9 (13)	O4—C9—C8	104.39 (17)
C3—C4—C5	102.07 (17)	N1—C9—C8	106.81 (17)
C3—C4—H5	108.5 (13)	O4—C9—H13	108.0 (11)
C5—C4—H5	113.0 (13)	N1—C9—H13	106.1 (11)
C3—C4—H6	110.0 (15)	C8—C9—H13	117.0 (11)
C5—C4—H6	113.8 (15)	O4—C10—C7	105.13 (18)
H5—C4—H6	109 (2)	O4—C10—H14	110.0 (15)
N1—C5—C4	105.22 (16)	C7—C10—H14	112.6 (15)
N1—C5—C6	112.30 (17)	O4—C10—H15	108.9 (13)
C4—C5—C6	116.70 (19)	C7—C10—H15	112.1 (13)
N1—C5—H7	106.4 (12)	H14—C10—H15	108 (2)
C4—C5—H7	107.7 (13)		
